# Adipose‐Derived Mesenchymal Stem Cell‐Derived Exosomes Biopotentiated Extracellular Matrix Hydrogels Accelerate Diabetic Wound Healing and Skin Regeneration

**DOI:** 10.1002/advs.202304023

**Published:** 2023-09-15

**Authors:** Yanling Song, Yuchan You, Xinyi Xu, Jingyi Lu, Xiajie Huang, Jucong Zhang, Luwen Zhu, Jiahao Hu, Xiaochuan Wu, Xiaoling Xu, Weiqiang Tan, Yongzhong Du

**Affiliations:** ^1^ Institute of Pharmaceutics College of Pharmaceutical Sciences Zhejiang University Hangzhou Zhejiang 310058 P. R. China; ^2^ Shulan International Medical College Zhejiang Shuren University Hangzhou Zhejiang 310015 P. R. China; ^3^ Department of Plastic Surgery Sir Run Run Shaw Hospital School of Medicine Zhejiang University Hangzhou Zhejiang 310016 P. R. China; ^4^ Department of Pharmacy Sir Run Run Shaw Hospital School of Medicine Zhejiang University Hangzhou Zhejiang 310016 P. R. China; ^5^ Innovation Center of Translational Pharmacy Jinhua Institute of Zhejiang University Jinhua 321299 P. R. China

**Keywords:** adipose‐derived mesenchymal stem cells, diabetic wounds, exosomes, extracellular matrix hydrogel, wound healing

## Abstract

Wound healing is an urgent clinical challenge, particularly in the case of chronic wounds. Traditional approaches to wound healing have limited therapeutic efficacy due to lengthy healing times, risk of immune rejection, and susceptibility to infection. Recently, adipose‐derived mesenchymal stem cell‐derived exosomes (ADSC‐exos) have emerged as a promising modality for tissue regeneration and wound repair. In this study, the development of a novel extracellular matrix hydrogel@exosomes (ECM@exo) is reported, which entails incorporation of ADSC‐exos into an extracellular matrix hydrogel (ECM hydrogel). This solution forms a hydrogel at physiological temperature (≈37 °C) upon local injection into the wound site. ECM@exo enables sustained release of ADSC‐exos from the ECM hydrogel, which maintains high local concentrations at the wound site. The ECM hydrogel displays good biocompatibility and biodegradability. The in vivo and in vitro results demonstrate that ECM@exo treatment effectively reduces inflammation and promotes angiogenesis, collagen deposition, cell proliferation, and migration, thereby accelerating the wound healing process. Overall, this innovative therapeutic approach offers a new avenue for wound healing via a biological hydrogel with controlled exosome release.

## Graphical abstract

In this research, the authors construct an injectable extracellular matrix (ECM) hydrogel by encapsulating exosomes derived from ADSCs, which can provide a sustained release of exosomes in the wound site. This strategy enhances cell proliferation, angiogenesis, collagen deposition, and reduces inflammation, which accelerates wound healing and remodels the normal physiological structure of the skin in normal and diabetic wound models.

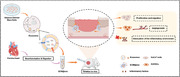

## 1 Introduction

As the largest organ of the human body, the skin is composed of various cells and maintains homeostasis.^[^ ^1^ ^]^ Wound healing is essential in maintaining and restoring epidermal barrier integrity after skin damage.^[^ ^2^ ^]^ In general, wound healing experiences four typical stages, including hemostasis, inflammation, proliferation, and tissue remodeling.^[^ ^3^ ^]^ However, wound healing of patients is affected by many factors, especially in patients with diabetes, hypertension, rheumatologic and inflammatory disease.^[^ ^4^, ^5^ ^]^ Taking diabetic wounds as an example, there are hundreds of millions of people living with diabetes worldwide. Approximately 10–25% of diabetic patients face slow wound healing or diabetic foot ulcers (DFUs).^[^ ^6^, ^7^ ^]^ Hyperglycemia‐induced microvascular dysfunction, peripheral neuropathy, and persistent inflammation may be the major reasons for delayed healing.^[^ ^8^, ^9^, ^10^ ^]^ Generally, clinical treatments for wound healing include surgical debridement, graft transplantation, wound dressing, physical hyperbaric oxygen therapy and so on. However, the disadvantages of these methods, such as long healing time, high cost, immune rejection, and easy infection, limit their application.^[^ ^11^, ^12^ ^]^ Therefore, new strategies of more effective treatments for wound healing, especially chronic wounds, are urgently needed for clinical application.

In recent years, mesenchymal stem cells (MSCs) have been confirmed to play an important role in tissue regeneration and wound repair.^[^ ^13^, ^14^ ^]^ Studies have found that MSCs can increase angiogenesis, regulate extracellular matrix (ECM) remodeling, and accelerate wound closure.^[^ ^15^, ^16^ ^]^ Compared to other kinds of MSCs, adipose‐derived mesenchymal stem cells (ADSCs) are widely available and can secrete most of the cytokines related to wound repair.^[^ ^17^ ^]^ Moreover, the effect of ADSCs therapy is realized through the paracrine effect mediated by ADSCs secretory factors, exosomes.^[^ ^18^, ^19^ ^]^ In comparison with ADSCs, exosomes have low immunogenicity, high stability, and easy storage.^[^ ^20^, ^21^ ^]^ Exosomes used for wound healing have gradually become a focus of research in recent years.^[^ ^22^ ^]^ It was reported that ADSC‐derived exosomes (ADSC‐exos) increased the migration, proliferation and blood vessel regeneration potential of vascular endothelial cells (ECs).^[^ ^23^ ^]^ Furthermore, other studies also confirmed that ADSC‐exos promoted macrophage M2 polarization, thereby enhancing both the proliferation and migration of fibroblasts and the angiogenesis of ECs.^[^ ^24^, ^25^ ^]^ A series of studies confirmed that extracellular vesicles (Evs) could prevent tissue damage, reduce inflammation and remodel vasculature,^[^ ^26^, ^27^ ^]^ and miR‐130a and Tgf β content correlated with EVs in‐vitro and in‐vivo angiogenic properties.^[^ ^28^ ^]^Moreover, a pilot clinical study recently demonstrated efficacy in using autologous serum derived Evs.^[^ ^29^ ^]^ However, employing exosomes to heal wounds still faces challenges. Exosomes cannot accumulate at the wound site in high concentrations for a long time due to their rapid clearance rate and relatively short half‐life in vivo.^[^ ^30^ ^]^ Therefore, constructing a biocompatible carrier capable of loading exosomes, maintaining the biological activity of exosomes, and achieving sustained release would be the key to applying exosomes to clinical wound healing.

Many studies have shown that hydrogels can provide a suitable repair environment in the process of wound healing and are considered promising drug carrier materials.^[^ ^31^, ^32^, ^33^ ^]^ For example, Ramírez et al. proved that EVs integrated into type I collagen hydrogels displayed the most stable release and promoted cell migration^[^ ^34^ ^]^ Yang et al. found that EV‐loaded hyaluronan hydrogel promotes scarless skin healing.^[^ ^35^ ^]^ Zhou et al. reported that ADSC‐exosomes encapsulated in pluronic F127 hydrogel promoted wound healing and regeneration.^[^ ^36^ ^]^ Zhao et al. constructed a polypeptide hydrogel loaded β‐exo, which could provide a sustained release of exosomes.^[^ ^37^ ^]^ These studies proved the effect of hydrogels on sustained release. However, these materials could not quite simulate the natural environment of normal skin tissue, which is suitable for cells growth. To better mimic the in vivo environment, we constructed an extracellular matrix (ECM) hydrogel derived from myocardium. ECM, as the direct survival environment of cells, is composed of collagens, glycosaminoglycans, proteoglycans, and so on.^[^ ^38^, ^39^ ^]^ Many studies have found that the extracellular matrix can degrade to form thermosensitive and in situ‐forming injectable hydrogels.^[^ ^40^, ^41^ ^]^ ECM hydrogel solution freely flows at 4 °C and forms a stable hydrogel at 37 °C. Moreover, ECM hydrogel has other advantages, including appropriate pore structure, sustained bioactive molecule release, excellent biocompatibility, biodegradation and sufficient binding sites.^[^ ^42^, ^43^ ^]^ In addition, ECM hydrogel can promote a constructive and site‐appropriate remodeling response in vivo.^[^ ^44^, ^45^ ^]^ Based on these advantages, ECM hydrogel was selected as the carrier of exosomes.

**Scheme 1:**
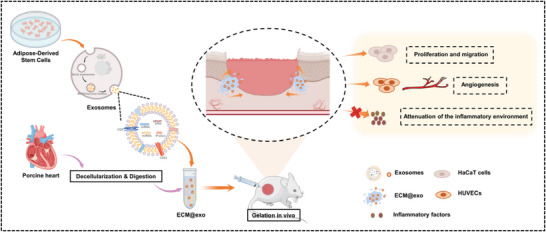
The preparation of ECM@exo and the role of ECM@exo in the wound healing process.

In this study, we constructed an injectable and thermosensitive hydrogel, extracellular matrix hydrogel@exosomes (ECM@exo), which exhibited sustained release of exosomes and excellent biocompatibility. ECM hydrogel was obtained from porcine left ventricular myocardium by decellularization and digestion, and ADSC‐exos were extracted from the supernatant of ADSCs by ultracentrifugation. ECM@exo was then prepared by thoroughly encapsulating ADSC‐exos into the ECM hydrogel. Once injected into the wound site, ECM@exo solution could form a hydrogel at physiological temperature (nearly 37 °C), as mentioned before. ADSC‐exos were slowly and continuously released from the hydrogel to maintain a high concentration in the wound site. At the same time, ECM hydrogel degraded gradually in vivo. Overall, we provide a promising strategy for exosome application in wound healing, which could effectively reduce inflammation and promote angiogenesis, collagen deposition, cell proliferation and migration, ultimately accelerating wound healing (**Scheme** **1**).

## 2 Results and Discussion

### 2.1 Isolation and Characterization of ADSCs and ADSC‐exos

**Figure 1:**
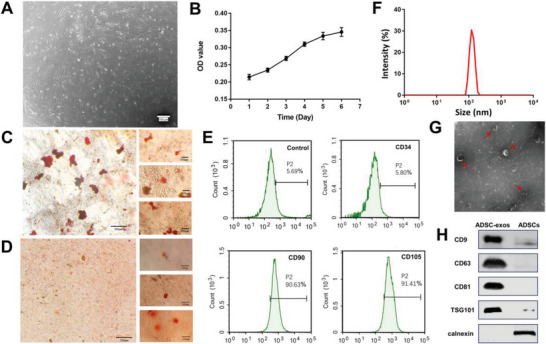
Characterization of ADSCs and ADSC‐exos. A) Cytomorphology of ADSCs after three‐passage cultivation. B) Growth curve of ADSCs within 6 days (*n* = 6). C) Adipogenic differentiation of ADSCs. Scale bar = 100 µm. D) Osteogenic differentiation of ADSCs. Scale bar = 100 µm. E) Analysis of ADSCs by flow cytometry (including CD90, CD105, CD34). F) Size distributions of ADSC‐exos by dynamic light scattering (DLS). G) Transmission electron microscopy (TEM) image of ADSC‐exos. Scale bar = 200 nm. H) Western blot (WB) of ADSC‐exos and ADSCs.n

As illustrated in **Figure** **1**A, ADSCs exhibited a classical fibroblast‐like appearance and plastic‐adherent properties after three passages of cultivation. The growth of ADSCs was normal, as shown by the MTT assay (Figure 1B). ADSCs still had the ability to differentiate into osteoblasts (Figure 1C) and adipocytes (Figure 1D). The results of flow cytometry analysis demonstrated that ADSCs highly expressed the surface markers CD90 and CD105 but expressed low levels of CD34 (Figure 1E). ADSC‐exos were obtained by ultracentrifugation, with an average particle size of 108.97±13.15 nm (Figure 1F). The ζ‐potential of exosomes was −8.63±2.25 mV, which was the same as the surface potential of the cell membrane. The TEM results showed that ADSC‐exos had a typical saucer‐like structure (Figure 1G). The WB results indicated that CD9, CD63, CD81, and TSG101 expression levels were significantly improved in ADSC‐exos, compared with ADSCs. Furthermore, calnexin was hardly found in ADSC‐exos (Figure 1H). These results confirmed that ADSCs were isolated and ADSC‐exos were extracted form cells supernatant via ultracentrifugation successfully.

### 2.2 Preparation and Characterization of Thermosensitive ECM Hydrogel

**Figure 2:**
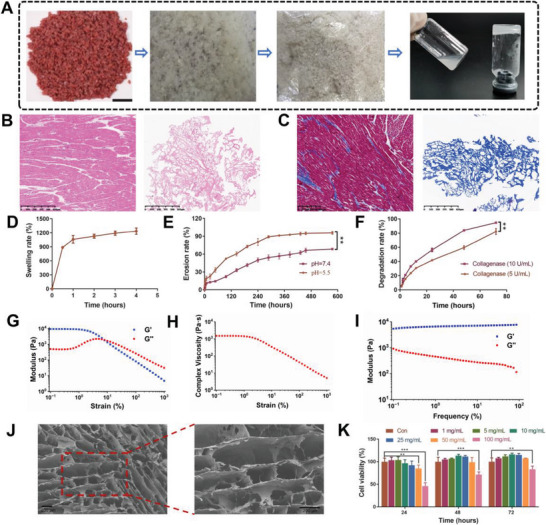
Preparation and characterization of ECM hydrogel. A) Schematic illustration of the preparation process of ECM hydrogel. B) Hematoxylin‐eosin (H&E) staining and C) Masson's trichrome staining of porcine cardiac tissue before and after decellularization. Scale bar = 125 µm. D) Swelling rate, E) erosion rate, and F) degradation rate of ECM hydrogel (*n* = 3). G) Rheology analysis of ECM hydrogel in strain sweep experiment. H) The viscosity of ECM hydrogel. I) Rheology analysis of ECM hydrogel in dynamic frequency sweep experiment. J) Scanning electron microscopy (SEM) cross‐sectional images of ECM hydrogel. Scale bar = 200 µm. K) Cytocompatibility of ECM hydrogel (*n* = 6). Differences among the groups were determined with one‐way ANOVA with Tukey's posttest. Data were considered statistically significant when *P < 0.05, **P < 0.01, and ***P < 0.001 versus the indicated group.

The extracellular matrix was successfully decellularized from porcine cardiac muscle (**Figure** **2**A). Hematoxylin‐eosin staining (H&E) and Masson's trichrome staining confirmed that the cells were almost completely removed and that nearly 38% of the ECM matrix was composed of collagen (Figure 2B). To screen suitable conditions for forming ECM hydrogel, the influences of particle size, ECM concentration, ionic strength, pH, and temperature were taken into consideration (Figures S1–S3, Supporting Information). Eventually, ECM digestion solution, with pH 7.4 and a concentration of 10 mg ml^−1^, could form a hydrogel in 2–3 min at 37 °C, and the ECM hydrogel formed in this condition was used for the following experiments. The results of the swelling test showed that the ECM hydrogel showed rapid swelling behavior in 4 hours, and the swelling rate reached nearly 1200% (Figure 2D). Degradability is essential to tissue engineering for hydrogel adhesives because they can be replaced by regenerative tissue. As illustrated in Figure 2F, the ECM hydrogel degraded rapidly in the presence of collagenase that exists naturally in the body. Approximately 95% of the ECM hydrogel degraded after 72 hours. However, the ECM hydrogel eroded slowly. After 25 days in PBS, the erosion rate reached nearly 95% at pH = 5.5 and 68% at pH 7.4, demonstrating hydrogel stability in the absence of enzymes (Figure 2E). The storage modulus (G′) represents the solid property of gel, and the loss modulus (G″) represents the liquid property of gel. Figure 2G showed that both G′ and G″ values were relatively stable in the strain range from 0.1% to 1%, which indicated the linear viscoelastic region of ECM hydrogel. What's more, the viscosity of ECM hydrogel remained steady in the range of 0.1–1% (Figure 2H). In Figure 2I, the storage modulus (G’) was substantially higher than the loss modulus (G″) with a frequency range of 0.1–100% Hz, which suggested the formation of an elastic hydrogel network. A more uniform porous structure provided more advantages in modulating drug release. The internal structures of the hydrogels were further analyzed by SEM (Figure 2J) to examine the porosity in the cross‐sectional area of the lyophilized hydrogels. It was revealed that ECM had a moderate porosity of ≈48% with an average pore size of 28 µm, which can provide more advantages in modulating drug release with a more uniform porous structure. A cytocompatibility test clarified that low concentrations of ECM hydrogel (1, 5, 10 and 25 mg ml^−1^) did not show any significant toxicity to HaCaT cells (Figure 2K). Instead, the low concentration could promote the growth of cells. These results demonstrated that the ECM hydrogel exhibited good cytocompatibility.

### 2.3 Exosome Release and Cell Proliferation, Migration, and Tube Formation

**Figure 3:**
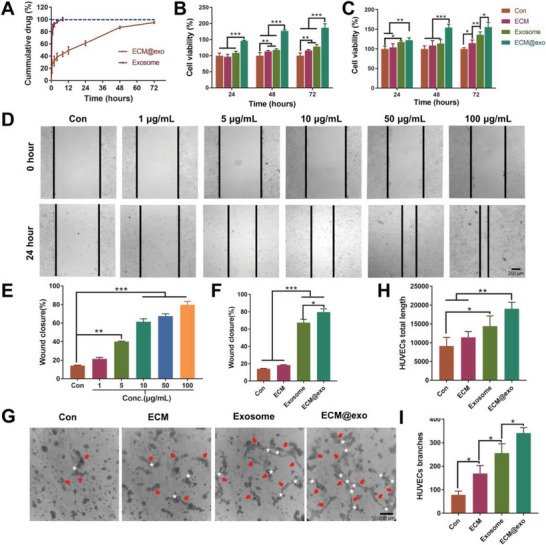
Exosome release and in vitro evaluation of ECM@exo. A) The release profile of exosomes from ECM@exo. B) HaCaT cells viability with different treatments (*n* = 6). C) HUVECs viability with different treatments (n = 6). B),C): the same figure legend. D) Images of HaCaT cells migration at 0 and 24 h. Scale bar = 200 µm. E) Statistical analysis of HaCaT cells migration treated with different concentrations of ECM@exo (n = 3). F) HaCaT cells migration with different treatments (n = 3). G) Images of HUVECs angiogenesis. Scale bar = 200 µm. (Main tube length marked by red arrow and branches marked by white star). Quantitative analysis of H) total length and I) branches, representing the tube formation ability of HUVECs with different treatments (*n* = 3). Differences among the groups were determined with one‐way ANOVA with Tukey's posttest. Data were considered statistically significant when *P < 0.05, **P < 0.01, and ***P < 0.001 versus the indicated group.

ADSC‐exos encapsulated in ECM hydrogel were released more slowly and continuously than free exosome solution (**Figure** **3**A). Evaluated by a BCA test kit, the release of ECM@exo reached 95.1±1.4% over 72 h, while the free exosome was released completely in 4 h. To explore the effects of ECM@exo on the behaviors of HaCaT cells and HUVECs, after coculture with ECM hydrogel, cell proliferation, migration, and tube formation were evaluated. The MTT assay was used to detect the proliferation of HaCaT cells and HUVECs at measurement time points. The experimental results indicated that the effect of promoting cell proliferation became more significant with increasing ECM@exo concentration, in both HaCaT cells and HUVECs (Figure S4A,B, Supporting Information). At the same time, compared with the ECM hydrogel group and free exosome group, the ECM@exo group showed a higher number of cells. The results indicated that continuously released extracellular vesicles can promote the proliferation of HaCaT cells and HUVECs (Figure 3B,C). In addition, the cell migration results confirmed that ECM@exo could significantly enhance the migration of HaCaT cells (Figure 3D–F). The tube formation assay showed that ECM@exo group displayed better tube formation performance with a greater total tube length and more tube branches than other groups, which indicated that ECM@exo could magnify angiogenesis in vitro (Figure 3G–I). These results showed that the ECM hydrogel had good biocompatibility, and the continuous release of exosomes from the ECM hydrogel could effectively promote cell proliferation, migration, and tube‐forming activities in vitro, which promoted wound healing. These results also suggested that ECM@exo had a potential promoting effect on wound healing.

### 2.4 In Vivo Wound Healing Evaluation of the Normal Wound Model

The wound healing ability of the ECM@exo hydrogel was examined using two kinds of full‐thickness cutaneous wound models, i.e., a normal wound healing model and a diabetic wound healing model.

**Figure 4:**
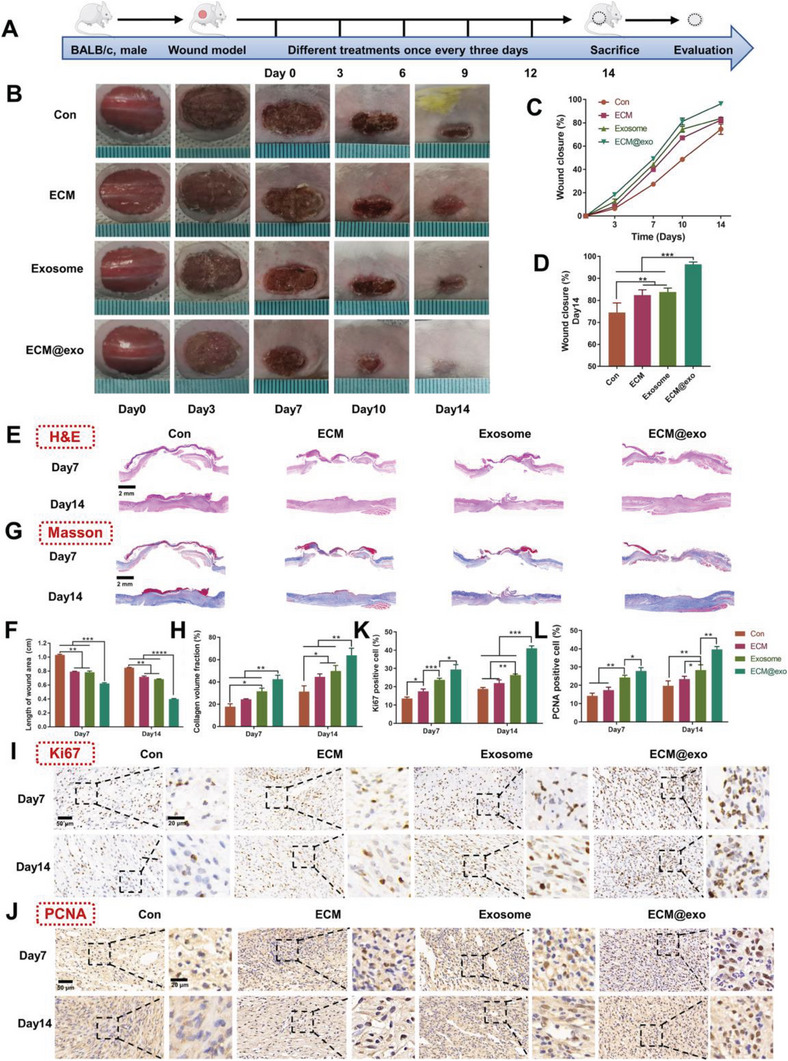
In vivo wound healing evaluation of the normal wound model. A) Schematic illustration of the animal model and the schedule of wound treatment in vivo. B) Images of the wound healing process of mice with different treatments. C,D) Wound closure rates of the four groups (*n* = 6). E) H&E staining images of wound samples at days 7 and 14. Scale bar = 2 mm. F) Quantitative analysis of the length of the wound area at day 7 and day 14 (*n* = 3). G) Masson staining images of wound samples at days 7 and 14. Scale bar = 2 mm. H) Quantitative analysis of collagen deposition at days 7 and 14 (n = 3). I) Images of immunohistochemical staining of Ki67, scale bar = 50 µm, and enlargement of the indicated area, scale bar = 20 µm. J) Images of immunohistochemical staining of PCNA, scale bar = 50 µm, and enlargement of the indicated area, scale bar = 20 µm. (K) and (L) are quantitative analysis of Ki67 and PCNA IHC staining, respectively (*n* = 3). F,H,K,L): the same figure legend. Differences among the groups were determined with one‐way ANOVA with Tukey's posttest. Data were considered statistically significant when *P < 0.05, **P < 0.01, and ***P < 0.001 versus the indicated group.

In the normal wound model, 15 mm full‐thickness skin wounds were created on the back of BALB/c mice, and these mice were randomly grouped and treated with ECM@exo, exosomes, ECM hydrogel, or PBS. Images of the wound were obtained at days 0, 1, 3, 7, 10, and 14. Meanwhile, during the healing process, the weight of the mice was continuously monitored, and the results showed that there was no significant difference in the weight of each group of mice (Figure S5, Supporting Information). After 14 days of treatment, the wound area of each group decreased, among which the wound closure rate of the ECM@exo group was the highest (**Figure** **4**B,C). Compared with the other groups, the wounds of mice in the ECM@exo group healed best, with a 96.4±0.9% closure rate on day 14, while the other groups reached final healing rates of 83.8±1.6% (exosome), 82.4±2.0% (ECM hydrogel) and 74.5±3.9% (control) (Figure 4D). On days 7 and 14, wound samples were collected and subjected to H&E staining and Masson staining to evaluate the histological status of the skin. As shown in Figure 4E, unlike the control group, which exhibited no formed neoepidermis, thick and abundant granulation tissue and new hair follicles were generated in the center of the wound of the ECM@exo group. The statistical data of wound length also confirmed that the ECM@exo group had the shortest lengths, followed by the exosome, ECM hydrogel and control groups (Figure 4F). These results indicated that ECM@exo both had good biocompatibility and could accelerate the healing process in the normal wound healin model. The Masson staining results revealed collagen deposition at the wound sites. At day 7, the wounds of mice in the control group had few collagen fibers and were only covered by eschar, whereas wounds treated with ECM@exo showed abundant and relatively well‐organized collagen fibers. The exosome and ECM hydrogel groups showed a moderate level of regenerated collagen. At day 14, newly formed collagen fibers were observed in all groups, but the ECM@exo group had the highest collagen volume fraction compared with the other groups. The ECM group and exosome group also showed certain therapeutic effects (Figure 4G,H). Masson staining results suggested that, as the exosome sustained release system, ECM@exo can improve collagen deposition and accelerate skin regeneration. Studies have proven that exosomes contain abundant microRNAs (miRNAs), which can promote the proliferation and migration of HaCaT cells via the PI3K/AKT pathway and Wnt/β‐catenin pathway.^[^ ^46^, ^47^ ^]^ Furthermore, miR‐128‐3p, miR‐125a‐3p and miR‐126‐3p have been reported to increase the viability, migration, and angiogenesis of HUVECs.^[^ ^48^, ^49^, ^50^ ^]^ In addition, it was also proven that ECM also obtained improved angiogenic capacity.^[^ ^51^ ^]^ Ki67, an important marker of cell proliferation, was also evaluated in wound samples at day 7 and day 14 to estimate total cellular proliferation. The immunostaining results of Ki67 showed that the expression of Ki67 increased significantly in the ECM@exo group compared with the other groups, while the control group had few positive cells (Figure 4I,K). Proliferating cell nuclear antigen (PCNA) is also a good indicator of cell proliferation status. The results of PCNA staining were similar to those of Ki67, where the ECM@exo group showed the highest expression of PCNA among all the groups, followed by the exosome group and ECM hydrogel group (Figure 4J,L). In summary, ECM@exo promoted cell proliferation during the healing period, which could explain the above granulation tissue formation and collagen deposition.

**Figure 5:**
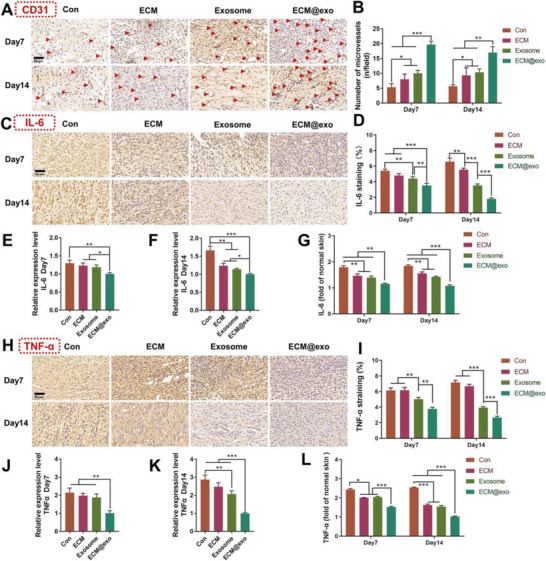
In vivo wound healing evaluation of the normal wound healing model. IHC Images A) and quantification results B) of CD31 at day 7 and day 14 (*n* = 3). Scale bar = 50 µm. IHC images (C) and quantification results (D) of IL‐6. Scale bar = 50 µm. qRT‒PCR quantitative analysis of IL‐6 at day 7 (E) and day 14 (F) (n = 3). G) Fold change of IL‐6 levels relative to those in normal skin tissue, as determined by ELISA. IHC images (H) and quantification results (I) of TNF‐α. Scale bar = 50 µm. qRT‒PCR quantitative analysis of TNF‐α. at day 7 (J) and day 14 (K) (*n* = 3). L) Fold change of TNF‐α levels relative to those in normal skin tissue, as determined by ELISA. Differences among the groups were determined with one‐way ANOVA with Tukey's posttest. Data were considered statistically significant when *P < 0.05, **P < 0.01, and ***P < 0.001 versus the indicated group.

Angiogenesis is an essential part of the entire process of wound healing. Blood vessels can boost cell proliferation and matrix remodeling at the wound site by providing progenitor cells, oxygen, and nutrients. CD31, as a marker of endothelial cells, was detected by IHC to assess the newly formed vessels. **Figure** **5**A,B show that the ECM@exo group had remarkable blood vessel numbers in contrast with the other groups. Inflammation is the second stage of cutaneous wound repair. Reducing the level of inflammation is beneficial for the wound to move to the proliferative stage. Studies have confirmed that miRNAs in exosomes have great potential in regulating the induction and regression of inflammatory responses by regulating the differentiation and development of immune cells and controlling the activation of inflammatory signaling pathways.^[^ ^52^, ^53^ ^]^ For example, miR‐34a‐5p, miR‐124‐3p, and miR‐146a‐5p induce the polarization of M2 macrophages, which results in attenuation of immune responses and inflammation.^[^ ^54^ ^]^ Tumor necrosis factor‐α (TNF‐α) and interleukin‐6 (IL‐6) were analyzed with IHC, qRT‒PCR and ELISA to explore the effect of ECM@exo on inflammation at the wound site. The IHC staining results showed that ECM@exo could reduce the expression of TNF‐α and IL‐6 so that the inflammatory response was alleviated (Figure 5C,D,H,I). The qRT‒PCR results also confirmed the same conclusion. (Figure 5E,F,J,K). In addition, we normalized the cytokine results using the concentration of inflammatory factors detected in normal skin. In comparison to other groups, ECM@exo significantly reduced the expression of IL‐6 and TNF‐α (Figure 5G,L). Furthermore, the ECM@exo content approximated natural levels by day 14 within the BALB/c mouse model. These results indicated that ECM@exo could reduce wound inflammation and facilitate wound concrescence.

### 2.5 In Vivo Wound Healing Evaluation of the Diabetic Wound Model

Under normal physiological conditions, cutaneous wounds can achieve self‐healing over a period of several days. However, for diabetic wounds, diabetic complications usually l to ischemia and delayed healing or even nonhealing, which results in adverse effects on patients’ life and health.^[^ ^8^, ^10^ ^]^ Therefore, effective treatments for accelerating the healing of chronic wounds have become a research focus. To explore the therapeutic effects of ECM@exo on diabetic wound healing, we established a diabetic wound model and conducted relevant experiments to evaluate the treatment effect. A diabetic mouse model was established successfully by injecting STZ after 5 weeks of hyperglycemia (Figure S6A, Supporting Information). During this period, ICR mice exhibited obvious polydipsia, polyphagia, and polyuria and gradually presented mental exhaustion and slow reaction. Unlike normal mice that could gradually gain weight, the weights of diabetic mice first increased with increasing feed intake and then decreased with the development of diabetes mellitus. (Figure S6B, Supporting Information).

**Figure 6:**
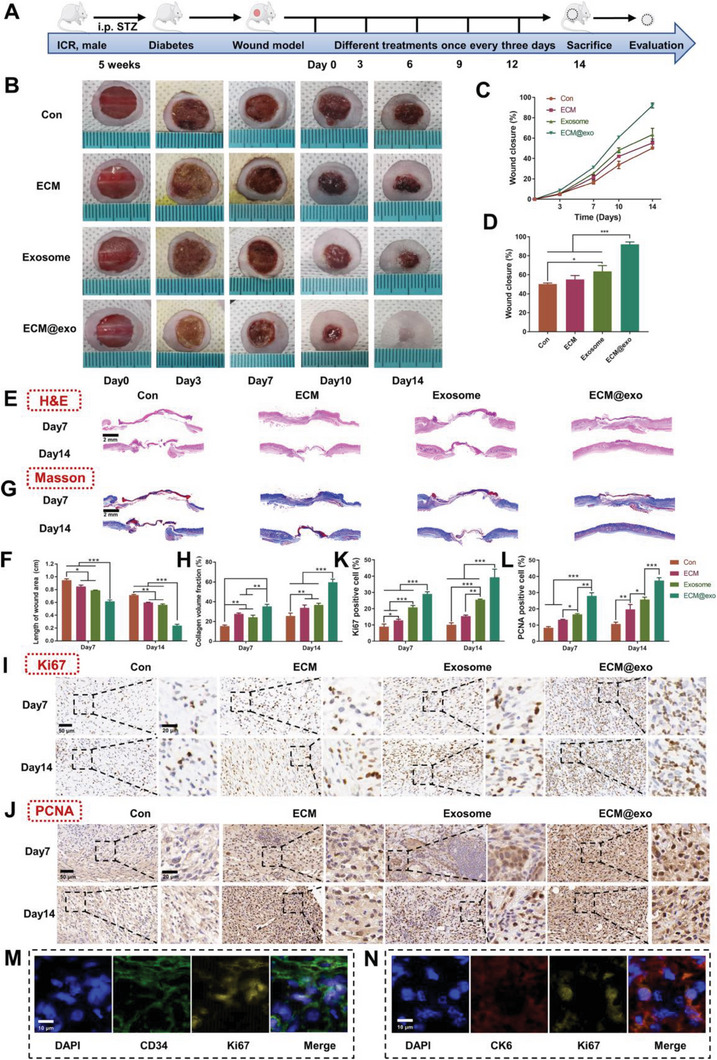
In vivo wound healing evaluation of the diabetic wound model. A) Schematic illustration of the animal model and the schedule of wound treatment in vivo. B) Images of the wound healing process of mice with different treatments. C,D) Wound closure rates of the four groups (*n* = 6). E) H&E staining images of wound samples at days 7 and 14. Scale bar = 2 mm. F) Quantitative analysis of the length of the wound area at day 7 and day 14 (*n* = 3). G) Masson staining images of wound samples at days 7 and 14. Scale bar = 2 mm. H) Quantitative analysis of collagen deposition at day 7 and day 14 (*n* = 3). I) Images of immunohistochemical staining of Ki67, scale bar = 50 µm, and enlargement of the indicated area, scale bar = 20 µm. J) Images of immunohistochemical staining of PCNA, scale bar = 50 µm, and enlargement of the indicated area, scale bar = 20 µm. (K) and (L) are quantitative analyses of Ki67 and PCNA IHC staining, respectively (*n* = 3). F,H,K,L): the same figure legend. M) Immunofluorescence with CD34 and Ki67 of wound samples. Scale bar = 10 µm. N) Immunofluorescence with CK6 and Ki67 of wound samples. Scale bar = 10 µm. Differences among the groups were determined with one‐way ANOVA with Tukey's posttest. Data were considered statistically significant when *P < 0.05, **P < 0.01, and ***P < 0.001 versus the indicated group.

**Figure 7:**
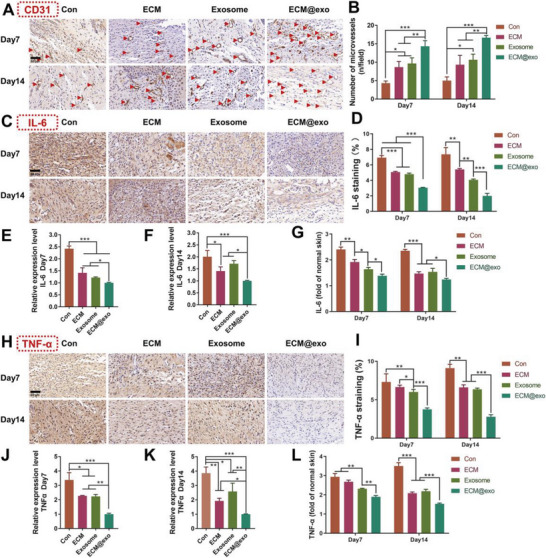
In vivo wound healing evaluation of the normal diabetic wound model. A,B) IHC images and quantification results of CD31 at day 7 and day 14. Scale bar = 50 µm. IHC images (C) and quantification results (D) of IL‐6. Scale bar = 50 µm. qRT‒PCR quantitative analysis of IL‐6 at day 7 (E) and day 14 (F) (*n* = 3). G) Fold change of IL‐6 levels relative to those in normal skin tissue, as determined by ELISA. IHC images (H) and quantification results (I) of TNF‐α. Scale bar = 50 µm. qRT‒PCR quantitative analysis of TNF‐α. at day 7 (J) and day 14 (K) (*n* = 3). L) Fold change of TNF‐α levels relative to those in normal skin tissue, as determined by ELISA. Differences among the groups were determined with one‐way ANOVA with Tukey's posttest. Data were considered statistically significant when *P < 0.05, **P < 0.01, and ***P < 0.001 versus the indicated group.

Full‐thickness skin wounds (10 mm) were created on the back of diabetic ICR mice, which were treated as the normal wound healing model. The collections of images and samples were also the same as the normal ones. No significant difference in weight was found during the healing period among the four groups. (Figure S7, Supporting Information). **Figure** **6**B illustrated that the wound closure rate of the control group was only 50% 14 days after surgery. The H&E and Masson staining results indicated that the control group had the longest wound length and lowest collagen deposition. These results showed that diabetic wounds were often difficult to heal without treatment (Figure 6E–H). ECM@exo had a positive effect on diabetic wound healing, with a 92% wound closure rate, abundant granulation tissue, and regenerated collagen at the wound sites, which showed that ECM@exo had a significant therapeutic effect on refractory wounds (Figure 6B‐H). Furthermore, studies have shown that hyperglycemia‐induced microvascular dysfunction may be one of the major causes of diabetic complications, resulting in ischemia and delayed healing in wounds. As shown in Figures 6I–L and **7**A,B through IHC staining, the ECM@exo group showed the highest expression of Ki67, PCNA, and CD31 among all the groups. These results showed that ECM@exo significantly promoted cell proliferation and angiogenesis. Moreover, double immunostaining results showed that CD34^+^/Ki67^+^ cells (Figure 6M) and CK6^+^/Ki67^+^ cells (Figure 6N) were found in the IF staining, which indicated that ECM@exo promoted both epithelial cells and endothelial cells proliferation in vivo.

Persistent inflammation was also an important reason for the delayed wound healing of diabetic mice, which resulted in the inability to transition between the inflammatory and proliferative stages.^[^ ^9^ ^]^ Compared with normal wounds, the inflammation level of diabetic wounds was even higher (**Figure** **7**C–L), which proved that persistent inflammation existed in the diabetic mice. The IHC, qRT‒PCR and ELISA results showed that ECM@exo could lower the inflammation level of diabetic wounds (Figure 7C–L). These results suggested that ECM@exo could accelerate wound healing, promote cell proliferation, stimulate angiogenesis, and reduce inflammation to achieve efficient and safe healing of chronic wounds.

## 3 Conclusion

In summary, we successfully fabricated a biologic ECM hydrogel loaded with ADSC‐exos. ECM@exo possessed many ideal properties, including thermosensitivity, injectability, appropriate pore structure, biodegradability, excellent biocompatibility, and sustained exosome release at the wound site. ECM@exo significantly improved the proliferation and migration of HaCaT cells, as well as the angiogenesis of HUVECs in vitro. Further in vivo studies indicated that ECM@exo enhanced cell proliferation and migration, angiogenesis, and collagen deposition and reduced inflammation, which accelerated wound healing and remodeled the normal physiological structure of the skin in normal and diabetic animal wound models. These results demonstrate that we provide a new strategy for more effective treatment of normal wound healing and chronic wound healing by loading exosomes into ECM hydrogel. Despite our findings, limitations in this study should be noted. For instance, pinpointing the specific activities associated with exosomal components in the context of wound healing and skin regeneration is critical, yet our study did not fully address this. Going forward, research should focus on elucidating these exact components and their impact on wound healing. Such an approach will bolster the potential for these findings to have superior clinical application.

## 4 Experimental Section

### Isolation, Culture, and Identification of ADSCs

Mouse testicular adipose tissues were acquired from healthy C57BL/6 mice.^[^ ^55^ ^]^ After washing with phosphate‐buffered saline (PBS) (Meilin, China) and fragmentation into ≈1 mm^3^ pieces, the adipose tissues were digested with 2 mg mL^−1^ collagenase type I (Worthington, USA) for 45 min. Then, an equal volume of complete culture medium (DMEM‐F12 culture medium (Solarbio, China), 10% FBS, 1% penicillin‒streptomycin) were added to terminate the digestion. After being filtered through a 70 µm mesh, the isolated cells were centrifuged for 5 min at 1500 rpm. Then, cells in Red Blood Cell Lysis Buffer (Beyotime, China) were resuspended for 2 min. After centrifugation and resuspension, the resultant cells were seeded in complete culture medium at 37 °C. When the adherent cells reached 80−90% confluence, the cells were passaged at 1:2 or 1:3. After three passages of cultivation, ADSCs could be used for subsequent experiments. Then, the cytomorphology of ADSCs was observed by optical microscopy. To identify the phenotypes of ADSCs, cells were examined for the expression of the surface markers CD34, CD90 and CD105 (Biolegend, USA) by flow cytometry. Osteogenic differentiation and adipogenic differentiation were induced by culturing ADSCs in a mouse adipose mesenchymal stem cell‐induced differentiation kit (Oricell, China). The differentiation into osteoblasts or adipocytes was examined by staining samples with alizarin red or Oil Red O staining.

### Isolation and Identification of ADSC‐Derived Exosomes

ADSC‐exos were isolated from cell supernatants as described previously^[^ ^56^, ^57^ ^]^ When ADSCs reached 80−90% confluence, the FBS‐free culture medium was changed. The culture supernatant at 3000 X g was centrifuged for 30 min to remove dead cells, followed by centrifugation at 10 000 X g for 30 min to eliminate cellular debris. After that, the remaining supernatant was filtered through a 0.22 µm filter to remove vesicles and apoptotic bodies. The clarified supernatant was ultracentrifuged at 100 000 X g for 90 min, and the exosome‐containing pellets were resuspended in PBS. The above operation procedures were performed at 4 °C, and the obtained exosomes were stored at −80 °C or used immediately. The average protein concentration of the exosome suspension was measured by a BCA quantitation kit (Beyotime, China). The particle size distribution and ζ‐potentials of exosomes were measured by dynamic light scattering (DLS). The isolated exosomes were characterized by transmission electron microscopy (TEM) for ultrastructural identification. Western blot was performed to evaluate the expression of ADSC‐exos positive markers CD9 (ABclonal, China), CD63(ABclonal, China), CD80 (Abcam, UK), and TSG101 (HuaBio, China), and the negative marker calnexin (ABclonal, China).

### Preparation and Characterization of Extracellular Matrix Hydrogel

Porcine cardiac tissue was collected and decellularized according to previously published protocols^[^ ^58^, ^59^ ^]^ Briefly, porcine hearts were obtained from a local slaughterhouse and dissected to separate the left myocardial tissue with the removal of epicardial adipose tissue, major vessels, and connective tissue. Then, the left ventricle was triturated in a commercial blender until small cubes with side lengths of 2 mm each were obtained. The tissue was washed at room temperature (RT) in ultrapure water for 60 min repeatedly. After that, the tissue was decellularized in 1% (wt/vol) sodium dodecyl sulfate (SDS) in PBS and 0.5% penicillin/streptomycin. The solution was changed daily for 5–7 days until the color of the tissue became white. Once decellularized entirely, the tissue was rinsed with ultrapure water to remove detergent. The tissue was then shaken in water, frozen at −80 °C, lyophilized and milled into a fine powder through a #40 sieve using a Wiley Mini Mill. The resulting powder was the extracellular matrix (ECM) material. To evaluate the extent of decellularization, tissue samples before and after decellularization were prepared for histochemical analysis. Then, cryosections were stained with hematoxylin and eosin (H&E). Then, cryosections were also stained with Masson's trichrome staining to analyze collagen expression. After decellularization, ECM material was enzymatically digested by pepsin (Sigma, USA). ECM material was added to a solution of pepsin in hydrochloric acid solution (1 mg/mL pepsin, 0.1 M HCl) so that the final concentration of ECM material was 10 mg mL^−1^. Then, the material was digested for 48–56 h at room temperature with constant stirring at 60–100 rpm. When no visible particles were found in the homogeneous liquid, the end‐production solution was adjusted to pH 7.4 by gradually adding 1 m sodium hydroxide (NaOH) and equilibrated the electrolytes with 10 X PBS, and all steps were performed on ice. The final ECM solution was kept at 4 °C for the following experiments. Standard conditions are defined as physiological conditions of pH 7.4, 1.0 X PBS, and 37 °C. Only one parameter (gelation temperature, gelation time, salt concentration or pH) at a time was varied from these standard conditions for any of the following experiments. ECM hydrogel was evaluated gel properties, including Swelling test, Erosion test, and In vitro degradation study (Procedures are described in the Supporting Information).

### Rheological Property Evaluation

The rheological properties of ECM hydrogel, including the storage modulus (G′) and loss modulus (G′′), were evaluated by a rheometer (MCR 302, Anton Paar, Austria). At 37 °C, the strain sweep was conducted in the range of 0.1–1000% strain at a frequency of 1 Hz, and the dynamic frequency sweep was performed in the range of 0.1–100% Hz at 1% strain.

### Microstructure Characterization

The microstructure of the ECM hydrogel was observed by scanning electron microscopy (SEM). First, the ECM hydrogel was prepared under standard conditions. Then, the sample was frozen and lyophilized. Finally, the lyophilized hydrogel sample was coated with a few nanometers (5–10 nm) of gold prior to SEM imaging.

### Cytocompatibility Test

The cytotoxicity of the ECM hydrogel was measured by MTT assay using human immortalized keratinocyte cells (HaCaT cells) (procedures are described in the Supporting Information).

### ECM@exo Preparation and Exosome Release Analysis

According to the protein concentration of the ADSC‐exos measured as described above, the ADSC‐exos were diluted to the target concentration. Then, the ECM solution and ADSC‐exo suspension were mixed to prepare the ECM@exo solution, and the final concentration of exosomes was 100 µg mL^−1^. Exosome release from the ECM@exo hydrogel was measured as previously described. Briefly, 500 µL of ECM@exo solution was added to the upper chamber of the Transwell insert (Labselect, China) in a 24‐well plate and incubated at 37 °C for gelation. Then, 1 mL PBS was added into the lower chamber as the dissolution medium, and free ADSC‐exo solution was the control group. At certain time points, 50 µL of PBS was collected, and the dissolution medium was replaced with fresh PBS. The exosome release from ECM@exo was detected by a micro‐BCA assay kit (Beyotime, China). The release content was calculated and expressed by the release percentage over time.

### Cell Proliferation, Migration, and Tube Formation Assay

In vitro studies, as described in the exosome release analysis, ECM@exo solution was added in the upper chamber of the Transwell insert and incubated at 37 °C for gelation. Cells were cultured on the lower chamber. At different time points after coculture with ECM@exo, cells were used for the following experiments.

### MTT Assay

To estimate the effect of ECM@exo on cell proliferation, HaCaT cells and HUVECs were treated with ECM@exo at different concentrations (exosome concentrations: 0, 1, 5, 10, 50, and 100 µg mL^−1^). After incubation for 24, 48, 72 h, the cell viability was measured by MTT assay. Moreover, the ability of different treatments to promote cell proliferation was also measured by MTT assay. Four groups were analyzed: 1) ECM@exo hydrogel group: ECM hydrogel containing 100 µg mL^−1^ exosome; 2) exosome group: 100 µg mL^−1^ free exosome solution; 3) ECM hydrogel group: blank ECM hydrogel; and 4) control group: PBS. The groups and treatments of the following experiments were the same.

### Scratch Assay

Cell migration was estimated using a scratch assay. In brief, HaCaT cells were cultured in the lower chamber of 12‐well plates at 1 × 10^5^ cells mL^−1^. When the HaCaT cells reached ≈95−100% confluence per well, a scratch was made using a 200 µL pipette tip. Four groups were analyzed as before. The width of the scratch was visualized with a light microscope, and the wound closure rate was measured by ImageJ software.

### Tube Formation Assay

In the tube formation assay, human umbilical vein endothelial cells (HUVECs) were used to investigate the tube formation of ECM@exo. HUVECs were cultured in RPMI‐1640 containing 10% FBS and 1% penicillin‒streptomycin. One hundred microliters of Matrigel basement membrane matrix was first added to 24‐well plates and placed in a cell incubator for 10 min to solidify. HUVECs at a density of 150 000 per well were seeded into the plates. Four groups were analyzed as before. After HUVECs were incubated for 4 h at 37 °C, tube formation was detected by using a microscope. Total length and branches were measured by ImageJ software.

### Mouse Cutaneous Wound Model and Treatment Strategy

All animal protocols were approved by the Institutional Animal Care and Use Committee of Zhejiang University in this study.

### Normal Wound Model

Healthy BALB/c mice (male, 6 weeks, 20–25 g) were used in this experiment. After anesthetization with 0.3% phenobarbital sodium (0.1 mL/10 g) through intraperitoneal injection and shaving dorsal hair with an electric clipper, the mice were depilated with depilatory cream on the back. Then, a 15‐mm full‐thickness cutaneous wound was created on the midline line of the mouse back.

### Diabetic Wound Model

Healthy ICR mice (male, 6 weeks, 20–25 g) were used in this experiment. The mice were intraperitoneally injected with 1% streptozotocin (STZ) (150 mg kg^−1^) after a 12 h fast to induce the type 1 diabetes model according to previous work.^[^ ^20^, ^60^ ^]^ STZ was dissolved in 0.1 M citrate‐sodium citrate buffer (pH 4.2–4.5). Blood glucose was measured with a blood glucose monitor 3 days later. Mice with a blood glucose level below 20 mM were considered failure models and injected with STZ once again. Mice with blood glucose levels higher than 20 mM for at least 5 weeks were defined as diabetic mice. As described for the normal model, a 10‐mm full‐thickness cutaneous wound was created on the midline line of the mouse's back.

### Treatment Strategy

The mice were randomly divided into four treatment groups: 1) ECM@exo hydrogel group: ECM hydrogel containing 100 µg mL^−1^ exosome; 2) exosome group: 100 µg mL^−1^ free exosome solution; 3) ECM hydrogel group: blank ECM hydrogel; and 4) control group: PBS. There were at least six mice in each experimental group. Each group of mice was treated with different treatments once every three days, with a dose of 100 µL per mouse. After treatment for 7 and 14 days, some of the mice were killed, and the full layer of back skin was taken for subsequent experiments.

### In Vivo Wound Healing Evaluation

*Wound Closure Rates*: Wound healing was monitored over a period of 14 days. At days 0, 3, 7, 10, and 14, photographic images of the wound were taken and used to analyze the wound area by ImageJ software. The wound closure rate was calculated using the following formula:

(1) $$\mathrm{Wound}\;\mathrm{closure}\;\mathrm{rate}\left(\%\right)=\left(S_{0}-S_{t}\right)/S_{0}\times 100\%$$

*S*_0_: wound area on day 0. *S*_t_: wound area on day T.

### Histological Analysis, Immunohistochemistry, and Immunofluorescence

Mice were sacrificed, and regenerated skin samples were excised and collected on days 7 and 14. For histological evaluation, tissue sections were deparaffinized and rehydrated followed by H&E and Masson's trichrome staining. The quantification of IHC was analyzed by Image‐Pro Plus software. To detect angiogenesis in the wound, the wound tissue sections were stained with CD31 for immunohistochemical staining. In addition, IHC staining of PCNA and Ki67 was also applied to detect changes in the wound microenvironment and level of cell proliferation. For Ki67 and PCNA, data were represented as the percentage of positive cell numbers divided by total cell numbers. To identify the proliferating cells, IF staining was used with Ki67 antibody, CD34 antibody and Cytokeratin 6 (CK6) antibody (ABclonal, China).

### Evaluation of Tissue Inflammation

At days 7 and 14, skin samples were collected for evaluation of tissue inflammation. To measure the content of IL‐6 and TNF‐α, the mRNA levels of IL‐6 and TNF‐α were detected using qRT‒PCR. Additionally, tissue sections were analyzed by IHC staining with IL‐6 and TNF‐α antibodies, and IHC results were represented as the mean density (IOD/Area) of stained regions. Moreover, the cytokine results were normalized using the concentration of inflammatory factors detected in normal skin, measured with ELISA kits (MEIMIAN, China).

### Statistical Analysis

Data from at least three individual experiments are listed as the mean ± standard deviation (mean ± SD). The statistical trends from different groups were analyzed by one‐way ANOVA with Tukey's posttest (GraphPad Prism 8.0). Data were considered statistically significant when *P < 0.05, **P < 0.01, and ***P < 0.001 versus the indicated group.

## Conflict of Interest

The authors declare no conflict of interest.

## Author Contributions

Y.S. conceived the project and designed the experiments, performed the experiments, analyzed the data, and edited the manuscript. Y.Y. designed experiments, analyzed data, and edited manuscript. X.X. performed the experiments and analyzed the data. J.L. performed the experiments and analyzed the data. X.H. performed the experiments and analyzed the data. J.Z. performed the experiments and analyzed the data. L.Z. and J.H. analyzed data. X.W. conceived the project and designed experiments. W.T.acquired funding. X.X. and Y.D. acquired funding. Y.D. edited manuscript and supervised.

## Supporting information

Supporting Information

## Data Availability

The data that support the findings of this study are available from the corresponding author upon reasonable request.
