# Supplementary material for: Adipose‐Derived Mesenchymal Stem Cell‐Derived Exosomes Biopotentiated Extracellular Matrix Hydrogels Accelerate Diabetic Wound Healing and Skin Regeneration
Source: Adv Sci (Weinh). 2023 Sep 15;10(30):2304023. doi: 10.1002/advs.202304023 (PMC10602544; doi:10.1002/advs.202304023)
Supplement: Supplementary file 1 — Supporting Information [file ADVS-10-2304023-s001.pdf]

ADVANCED SCIENCE
Open Access

Supporting Information

for *Adv. Sci.*, DOI 10.1002/advs.202304023

Adipose-Derived Mesenchymal Stem Cell-Derived Exosomes Biopotentiated Extracellular Matrix Hydrogels Accelerate Diabetic Wound Healing and Skin Regeneration

Yanling Song, Yuchan You, Xinyi Xu, Jingyi Lu, Xiajie Huang, Jucong Zhang, Luwen Zhu, Jiahao Hu, Xiaochuan Wu, Xiaoling Xu, Weiqiang Tan* and Yongzhong Du**

Supporting information

Adipose-derived mesenchymal stem cell-derived exosomes biopotentiated extracellular matrix hydrogels accelerate diabetic wound healing and skin regeneration

Yanling Song[a], Yuchan You[a], Xinyi Xu[a], Jingyi Lu [a], Xiajie Huang[a], Jucong Zhang[a], Luwen Zhu[a], Jiahao Hu[a], Xiaochuan Wu[a], Xiaoling Xu [b], Weiqiang Tan [c], Yongzhong Du [a, c, d,e*]

[a] Institute of Pharmaceutics, College of Pharmaceutical Sciences, Zhejiang University, Hangzhou, Zhejiang 310058, P.R. China.

[b] Shulan International Medical College, Zhejiang Shuren University, Hangzhou, Zhejiang 310015, P. R. China,

[c] Department of Plastic Surgery, Sir Run Run Shaw Hospital, School of Medicine, Zhejiang University, Hangzhou, Zhejiang 310016, P.R. China.

[d] Department of Pharmacy, Sir Run Run Shaw Hospital, School of Medicine, Zhejiang University, Hangzhou, Zhejiang 310016, P.R. China.

[e] Innovation Center of Translational Pharmacy, Jinhua Institute of Zhejiang University, Jinhua 321299, P.R. China.

[*]Corresponding author. Yongzhong Du: duyongzhong@zju.edu.cn; Xiaoling Xu: ziyao1988@zju.edu.cn; Weiqiang Tan: tanweixxxx@163.com.

Experimental Section

Preparation and characterization of extracellular matrix hydrogel

Swelling test. The swelling property of ECM hydrogel samples was characterized by the gravimetric method. The lyophilized hydrogel samples were prepared in advance and weighed as the initial mass W_0. Then, the samples were immersed in ultrapure water at 37°C. At predetermined time intervals, we removed the samples and removed excess water on the surface using filter paper, followed by weighing them as W_t. When the mass of swollen hydrogels did not change over time, the samples reached the equilibrium swelling ratio. The swelling ratio was determined by the following equation.

$$\text{Swelling ratio (\%)} = (W_0 - W_t)/W_t \times 100$$

Erosion test. The erosion property of ECM hydrogels was determined by adding PBS (pH=7.4 and 5.5). The fresh hydrogel samples were weighed and described as W_0. Then, the hydrogels were immersed in 1 mL PBS and placed in a shaker at 37°C with a speed of 60 rpm. At each measurement time point, PBS was removed, and the weight was recorded as W_t until the hydrogel dissolved completely. The erosion ratio was calculated according to the following formula.

$$\text{Erosion ratio (\%)} = (W_0 - W_t)/W_t \times 100$$

In vitro degradation study. To measure the enzymatic degradation property of the ECM hydrogel, the hydrogel samples were placed in 5 U/mL and 10 U/mL collagenase type I solution (Worthington, USA) and incubated at 37°C with a speed of 60 rpm. At the desired timepoint, the hydrogel samples were removed from the degradation buffer, blotted dry and weighed as W_t. W_0 is the initial weight. The degradation percent of the ECM hydrogel was computed by using the formula below.

$$\text{Degradation percent (\%)} = (W_0 - W_t)/W_t \times 100$$

Cytocompatibility test. The cytotoxicity of the ECM hydrogel was measured by MTT assay using human immortalized keratinocyte cells (HaCaT cells). Typically, the complete culture medium of HaCaT cells consisted of Dulbecco's modified Eagle's medium (DMEM), 10% fetal bovine serum (FBS) and 1% penicillin–streptomycin. HaCaT cells at a density of 50000 cells/mL were cultured in 96-well plates and incubated overnight at 37°C with 5% CO_2. Then, the cell culture medium was removed and replaced with 200 μL fresh medium containing various quantities of ECM hydrogel (0, 1, 5, 10, 25, 50, 100 mg/mL). After incubation for 24, 48, 72 h, 20 μL of 5 mg/mL MTT solution was added to each well for 4 h at 37°C. Then, the culture medium was removed, and 150 μL dimethyl sulfoxide (DMSO) was used to dissolve the purple formazan crystals for 15 min with constant shaking. The result was measured at a wavelength of 562 nm. The calculation of cell viability was performed with the

following formula.

$$\text{Cell viability (\%)} = (A_t - A_b)/(A_c - A_b) \times 100$$

A_t and A_c are the OD values for cells cultured in hydrogel medium and normal medium, respectively. A_b is the OD value for the blank group.

Supplementary data

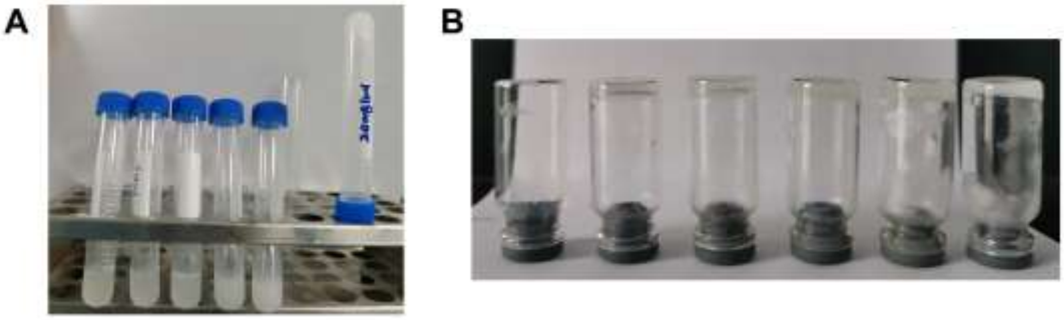

Figure S1 The influence of different concentrations on ECM hydrogel formation. A) ECM solution with different concentrations. B) ECM hydrogel formation with different concentrations.

From left to right, the concentrations were 5 mg/mL, 8 mg/mL, 10 mg/mL, 12 mg/mL, 15 mg/mL, and 20 mg/mL. These results indicated that the 5 mg/mL ECM solution could not form a hydrogel, and the 20 mg/mL ECM solution was too sticky to flow freely. 8 mg/mL ECM solution could form a gel in 3 min at 37°C with poor mechanical strength. ECM solutions (10, 12, and 15 mg/mL) could form hydrogels in 2 min at 37°C with good mechanical strength. Eventually, we chose 10 mg/mL ECM solution for the following experiments to save materials.

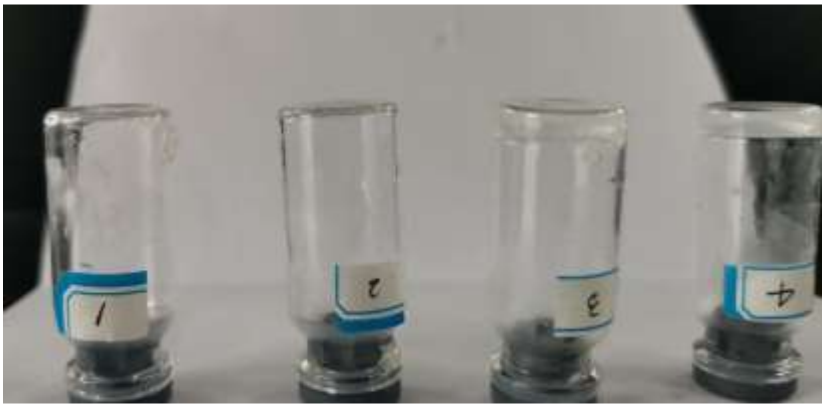

Figure S2 The influence of pH and ionic strength on ECM hydrogel formation. From left to right, group 1): digestion solution; group 2): digestion solution with physiological salt concentration (1X PBS); group 3): digestion solution with pH=7.4; group 4): digestion solution with pH=7.4 and physiological salt concentration (1X PBS). Groups 3 and 4 formed hydrogels, while groups 1 and 2 remained liquid. These results showed that pH had a greater effect on ECM hydrogel formation than ionic strength.

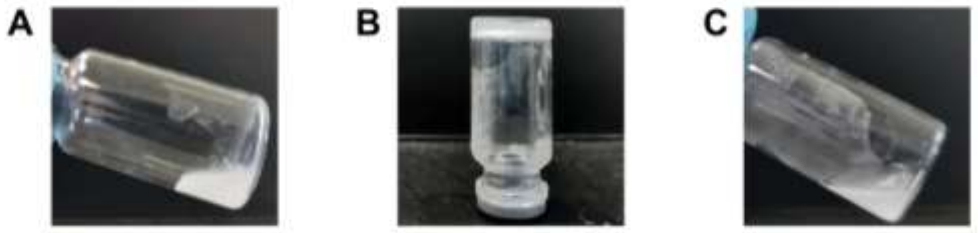

Figure S3 The influence of temperature on ECM hydrogel formation. A) ECM solution at 25°C. group B) ECM solution at 37°C. C) ECM solution at 45°C.

The ECM solutions at 25°C and 45°C were liquid, while the ECM solution at 37°C formed a hydrogel. These results confirmed the temperature sensitivity of the ECM hydrogel. It could not form a hydrogel at 45°C, which may be caused by protein denaturation at higher temperatures.

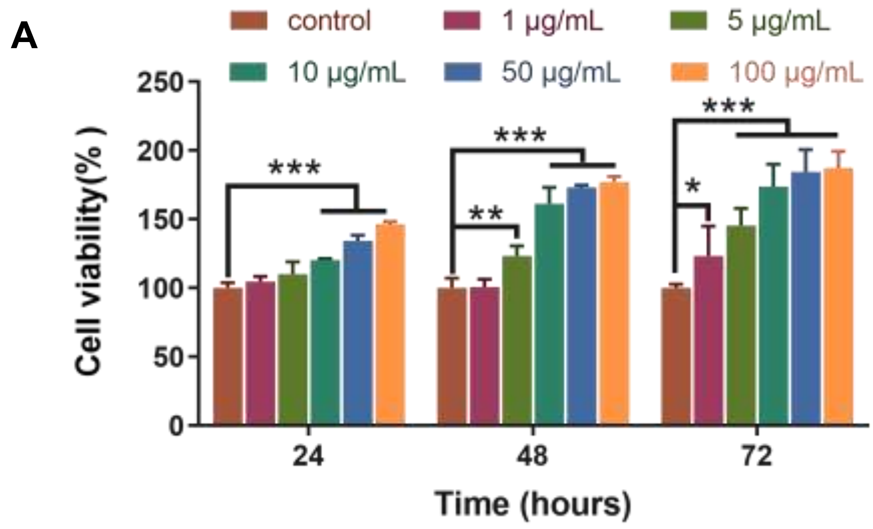

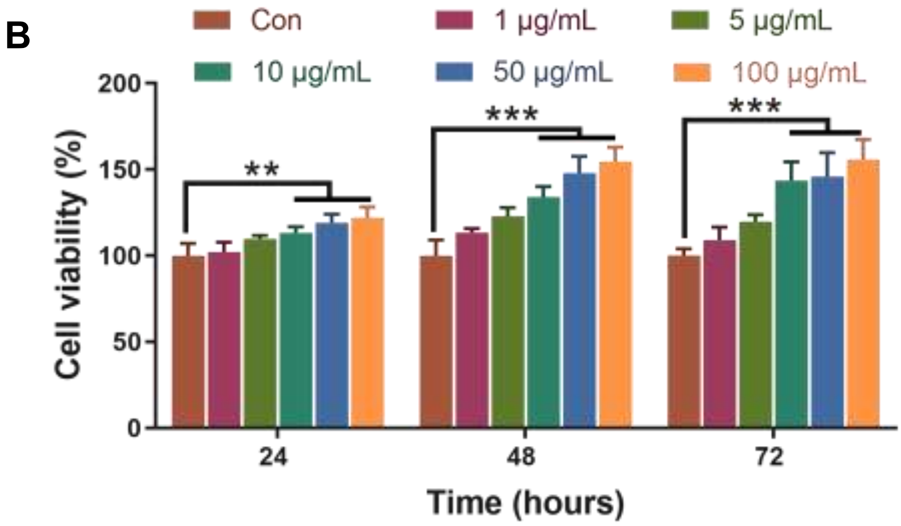

Figure S4 A) Viability of HaCaT cells treated with different concentrations of ECM@exo (n=6). B) Viability of HUVEC cells treated with different concentrations of ECM@exo (n=6).

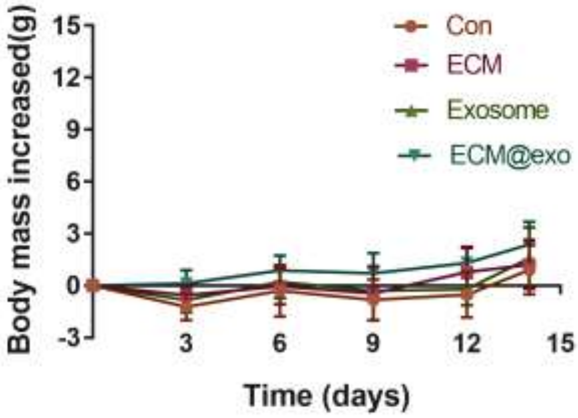

Figure S5 Weight changes in BALB/c mice with different treatments for 14 days.

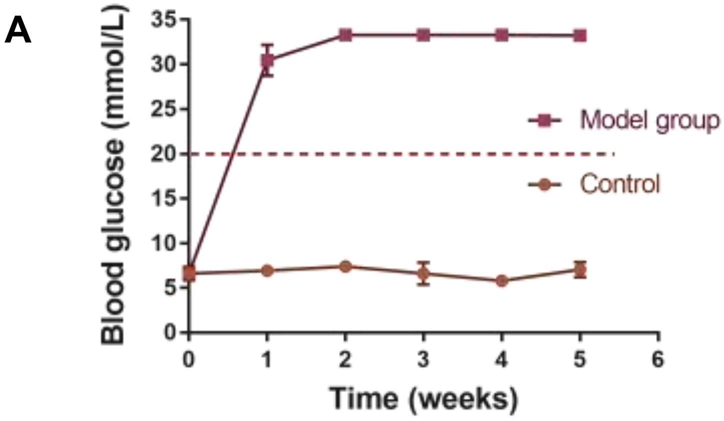

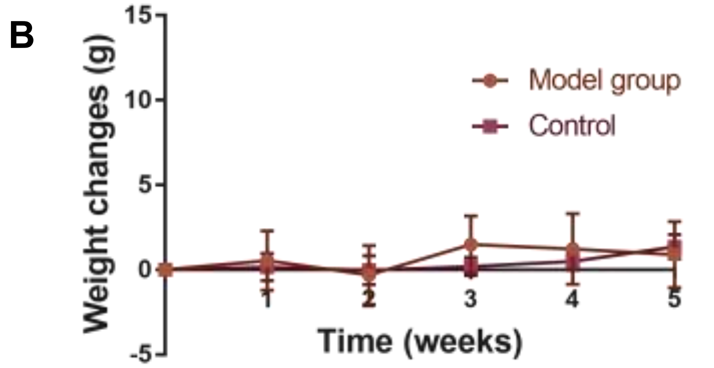

Figure S6 Diabetic wound healing model and weight changes. A) Blood glucose concentration of the Model group and Control group for 5 weeks. B) Weight changes in the model group and control group over 5 weeks.

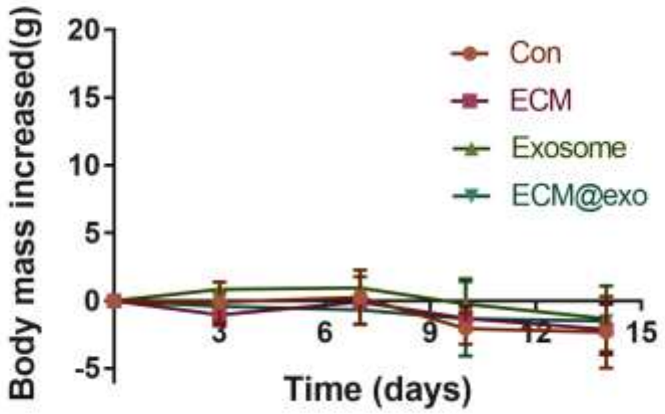

Figure S7 Weight changes of diabetic ICR mice with different treatments for 14 days.
